# Combustion and Heated Tobacco Cigarettes, but Not E-Cigarettes, Impair Aquaporin-Dependent H_2_O_2_ Permeability in ATII-Like Cells

**DOI:** 10.3390/cells15121112

**Published:** 2026-06-19

**Authors:** Giorgia Senise, Francesca Bodega, Cristina Porta, Umberto Laforenza

**Affiliations:** 1 Human Physiology Unit, Department of Molecular Medicine, University of Pavia, 27100 Pavia, Italy; giorgia.senise01@universitadipavia.it; 2 Dipartimento di Fisiopatologia Medico-Chirurgica e dei Trapianti, Università degli Studi di Milano, 20133 Milan, Italy; francesca.bodega@unimi.it (F.B.); cristina.porta@unimi.it (C.P.); 3 Centre for Health Technologies (CHT), University of Pavia, 27100 Pavia, Italy

**Keywords:** traditional cigarette smoke, heated tobacco, e-cigarette, peroxiporins, HyPer7 biosensor, oxidative stress, hydrogen peroxide, catalase, superoxide dismutase, glutathione peroxidase

## Abstract

**Highlights:**

**What are the main findings?**

- Cigarette smoke extract and, to a lower extent, heated tobacco product extract dramatically decrease the hydrogen peroxide permeability at both the plasma and at nuclear membranes.
- The diffusion of H_2_O_2_ through the inner mitochondrial membrane was blocked only by cigarette smoke extract.

**What are the implications of the main findings?**

- These results provide experimental evidence of the drastic reduction in H_2_O_2_ diffusion by cigarette smoke.
- Alternatives to conventional cigarettes show reduced damage compared with traditional cigarette smoke.

**Abstract:**

Cigarette smoke is a major inducer of oxidative stress, promoting reactive oxygen species (ROS) accumulation and contributing to the pathogenesis of chronic obstructive pulmonary disease (COPD) and lung cancer. Heated tobacco products (HTP) and e-cigarettes are promoted as reduced-risk alternatives; however, their impact on cellular redox regulation remains unclear. Here, we investigated the effects of conventional cigarette smoke extract (CSE), HTP, and e-cigarette extracts on hydrogen peroxide (H_2_O_2_) permeability mediated by aquaporins (peroxiporins) and on the activity of key antioxidant enzymes (catalase, superoxide dismutase, and glutathione peroxidase) in ATII-like cells. Eight aquaporins were detected at the mRNA level, and seven were confirmed at the protein level. CSE markedly inhibited H_2_O_2_ permeability across plasma, mitochondrial, and nuclear membranes. HTP extract impaired H_2_O_2_ transport across the plasma membrane and nuclear envelope, while mitochondrial permeability was preserved. Both CSE and HTP extract reduced superoxide dismutase and glutathione peroxidase activities. In contrast, e-cigarette extract exerted minimal effects on membrane H_2_O_2_ permeability and selectively decreased superoxide dismutase activity. Overall, our findings identify a graded pattern of oxidative toxicity (CSE > HTP > e-cigarette) and highlight peroxiporins as critical regulators of intracellular redox homeostasis. Although less harmful than cigarettes, alternative nicotine delivery systems are not biologically inert.

## 1. Introduction

Traditional combustible cigarette smoking causes widespread cellular damage through a range of interconnected mechanisms, including the generation of reactive oxygen species (ROS), exposure to toxic and carcinogenic compounds, and the activation of inflammatory pathways, which collectively contribute to oxidative stress, DNA damage, cellular senescence, and cell death [1]. These mechanisms are central to the development of smoking-related diseases, including chronic obstructive pulmonary disease (COPD), cardiovascular disease, and cancer [1].

Over the years, several strategies have been developed to reduce or stop traditional cigarette smoking. A wide range of behavioral, pharmacological, and policy-level interventions has been shown to support smoking reduction and cessation. Among these approaches, harm reduction strategies based on alternative nicotine delivery products have gained increasing attention. Nicotine replacement therapies and electronic cigarettes (e-cigarettes) can reduce cigarette consumption and may facilitate smoking cessation, although evidence regarding e-cigarettes is still emerging [2,3,4,5]. Similarly, nicotine reduction in cigarettes and the use of alternative nicotine delivery systems may decrease dependence and support quitting [3,6].

More recently, heated tobacco products (HTPs), also known as heat-not-burn products, have been marketed as alternatives to conventional cigarettes with claims of reduced harm. Current evidence indicates that HTPs generally induce lower cytotoxic and genotoxic effects than traditional cigarette smoke extract (CSE); however, they are not risk-free and still cause significant cellular damage. Several in vitro studies have demonstrated that HTP aerosols are less cytotoxic to human lung and oral cells than conventional cigarette smoke, but more toxic than e-cigarettes or air controls [7]. The cytotoxicity associated with HTP exposure is mediated by increased ROS production, altered calcium signaling, and inhibition of DNA repair, mechanisms similar to those induced by conventional cigarettes, albeit often at lower intensity [8,9].

Thus, increased ROS production emerges as one of the principal mechanisms underlying the cytotoxicity of both traditional cigarette smoke and HTP. Among ROS, hydrogen peroxide (H_2_O_2_) is the most abundant intracellular species, and its concentration is tightly regulated by multiple antioxidant systems, including peroxiporins.

Peroxiporins are a specialized subgroup of aquaporins (AQPs) that facilitate the diffusion of H_2_O_2_ across cellular membranes. By regulating H_2_O_2_ flux, peroxiporins play a pivotal role in maintaining redox homeostasis, modulating oxidative stress responses, and influencing pathological processes such as inflammation, cancer, neurodegeneration, and metabolic disorders. The peroxiporin family includes AQP0, AQP1, AQP3, AQP5, AQP6, AQP7, AQP8, AQP9, and AQP11 [10,11,12,13,14,15,16,17], although it has been suggested that most AQPs display some degree of H_2_O_2_ permeability [18].

By enabling the controlled movement of H_2_O_2_—an ROS involved in physiological signaling at low concentrations and in oxidative damage at high concentrations—peroxiporins allow cells to finely regulate redox signaling and prevent excessive ROS accumulation. In this context, peroxiporins function as a non-enzymatic antioxidant system, distinct from classical enzymatic defenses such as catalase or glutathione peroxidase, and can be considered key gatekeepers of cellular redox balance.

While AQP-mediated H_2_O_2_ permeability is essential for limiting oxidative stress in normal cells, increased AQP permeability in cancer cells may promote survival and contribute to resistance to conventional chemotherapeutic agents [13]. Furthermore, cellular stress conditions—including heat stress, hypoxia, and endoplasmic reticulum (ER) stress—have been shown to modulate the permeability of peroxiporins, thereby affecting cell growth and survival [15]. Such autoregulatory modulation of H_2_O_2_ permeability may represent a shared feature among different members of the peroxiporin family.

The aim of this study was to compare, using an in vitro cellular model of type II pneumocytes (ATII-like cells), the effects of traditional cigarette smoke, HTPs, and e-cigarette extracts on peroxiporin function. Specifically, we investigated the following in ATII-like cells: (1) AQP expression at both transcript and protein levels; (2) AQP subcellular localization; (3) the effects of CSE, HTP, and e-cigarette extract exposure on H_2_O_2_ permeability across the plasma membrane, the nuclear membrane, and the inner mitochondrial membrane; and (4) the impact of these treatments on the major cellular antioxidant enzymes.

The results presented herein highlight the profound toxic effects of conventional cigarette smoke extract, which markedly impairs AQP-mediated H_2_O_2_ diffusion. Notably, CSE exposure nearly abolishes H_2_O_2_ diffusion from the cell and severely restricts its transfer from mitochondria to the cytosol, leading to mitochondrial damage. Both CSE and HTP extract impair H_2_O_2_ permeability through the nuclear envelope. In general, HTP extract also exerts a detrimental effect on AQP-mediated H_2_O_2_ permeability, although to a lesser extent than conventional cigarettes. Finally, e-cigarette extract exerts minimal effects on the antioxidant function of AQPs, suggesting that e-smoking may represent a viable harm-reduction strategy for decreasing traditional cigarette consumption.

## 2. Materials and Methods

### 2.1. Cell Culture, Differentiation, and Treatments

A549 cells (CCL-185, American Type Culture Collection (ATCC) Manassas, VA, USA) were maintained at 37 °C in a humidified atmosphere containing 5% CO_2_ in Ham’s F12 Nutrient Medium (Merck, Milan, Italy) supplemented with 10% fetal bovine serum (Euroclone, Milan, Italy), 1% L-glutamine, 1% penicillin, and 100 µg/mL streptomycin (Merck, Milan, Italy). Culture medium was refreshed every 2–4 days.

To induce differentiation toward an ATII-like phenotype we followed the protocol optimized by Cooper et al. [19], consisting in culturing cells long-term (23–27 days) in Ham’s F12 instead of standard DMEM. Cooper et al. showed that, under these conditions, A549 cells exhibit gene expression and phenotypic features closer to primary ATII cells, including downregulation of proliferation-related genes and increased expression of pathways associated with differentiation, autophagy, and lipid metabolism [19]. We confirmed the reliability of the protocol of Cooper et al. in a previous work [20] showing, by TEM microscopy, that these cells had typical features of ATII, such as numerous cytoplasmic protrusions, a large rough endoplasmic reticulum, and many multilamellar bodies. Differentiation of A549 cells into ATII-like cells was confirmed by real-time RT-PCR analysis of mRNA expression of the alveolar markers alkaline phosphatase (ALP) and surfactant protein C (SP-C). As shown in Figure S1, both ALP and SP-C were expressed in ATII-like cells but were undetectable in undifferentiated A549 cells.

Cigarette smoke extract (CSE), heated tobacco product (HTP) extract, and e-cigarette extract were generated according to previously reported procedures with minor adaptations [20]. Briefly, smoking/vaping was performed using a 50 mL manually operated syringe with the following parameters: 50 mL puff volume, 2 s puff duration, and 30 s intervals. The resulting smoke/aerosol was bubbled into 10 mL of complete Ham’s F12 medium, and particulate matter was removed by filtration through a 0.45 μm syringe filter.

For CSE preparation, ten puffs from one cigarette (Marlboro, Philip Morris, Richmond, VA, USA) were dissolved in medium to obtain a 100% stock solution. The HTP device used was gloTM Hyper X2 (British American Tobacco, London, UK) with neo Terracotta Tobacco sticks; extracts were obtained by smoking each stick until automatic device shutdown (approximately 10–12 puffs). The device was operated in standard mode (maximal temperature 240 °C). For e-cigarettes, a Geekvape Soul Pod Kit (30 W, 1500 mAh; Shenzhen, China) equipped with a 0.6 Ω pod (25 W) was used with a liquid containing 50% propylene glycol, 50% glycerol, and 8 mg/mL nicotine; 15 puffs were bubbled into 10 mL medium to generate the extract. The device was operated at 25 W with a 0.6 Ω coil, corresponding to typical aerosolization conditions reported for similar systems. To minimize the loss of condensable components, the syringe and associated tubing used for e-cigarette and HTP extract were flushed with medium after puff collection to recover deposited material. Although partial condensation of aerosol components may occur during collection, bubble-through methods using aqueous media are widely employed for e-cigarette and HTP aerosol collection in in vitro studies [21].

HTP and e-cigarette extracts were applied at full strength (100%), whereas CSE was diluted to 60% in Ham’s F12 before use. Cells were exposed to treatments for 3 h at 37 °C in a CO_2_ incubator, while control samples received fresh medium only.

At the concentrations employed, no significant changes in cell number or viability were observed (Figure S2).

### 2.2. RNA Isolation, RT-PCR, and qPCR

Total RNA was extracted from A549 and ATII-like cells using QIAzol reagent (Qiagen, Milan, Italy) following the manufacturer’s guidelines. First-strand cDNA was generated using M-MLV Reverse Transcriptase (M1701; Promega, Milan, Italy). PCR amplification was carried out using Hot Disco Taq 2× Master Mix (Cat. No. 257685; Biosigma, Venice, Italy) with gene-specific primers listed in Table S1, as previously reported [22]. qPCR was performed on the Applied Biosystems StepOnePlus™ Real-Time PCR System (Thermo Fisher Scientific, Monza, Italy). qPCR was performed with an initial denaturation at 95 °C (15 min), followed by 40 cycles of 95 °C (30 s), 60 °C (30 s), and 72 °C (30 s). Melt curve analysis confirmed the presence of a single amplicon and no primer dimers.

Experimental details including primer sequences, annealing temperatures, and amplicon sizes are summarized in Table S1. β-actin served as the internal control. Amplified products were separated on 3% agarose gels containing ethidium bromide and visualized using the iBright™ CL1000 Imaging System (Thermo Fisher Scientific, Monza, Italy). Band sizes were estimated using DNA molecular weight marker VIII (11336045001; Merck, Milan, Italy). Gene expression was expressed as ΔCt, calculated by subtracting the Ct of the housekeeping gene (β-actin) from the Ct of the gene of interest. Notably, lower ΔCt values represent higher transcript expression values.

### 2.3. Western Blot Analysis

Protein extracts were obtained by lysing ATII-like cells in RIPA buffer (150 mM NaCl, 0.5% sodium deoxycholate, 0.1% SDS, 0.1% Triton X-100, 50 mM Tris-HCl, pH 8.0) supplemented with protease inhibitors (cOmplete™, 04693116001; Merck, Milan, Italy). Samples were mixed with Laemmli buffer, denatured at 95 °C for 5 min, and 30 μg protein per lane was resolved by SDS-PAGE using 4–15% gradient gels (Bio-Rad, Segrate, Italy).

Proteins were electrotransferred onto PVDF membranes using a Trans-Blot Turbo system (Bio-Rad Segrate, Italy). Membranes were blocked for 2 h at room temperature in TBS containing 5% non-fat dry milk and 0.1% Tween-20, followed by overnight incubation at 4 °C with primary antibodies (Table S2). β2-microglobulin was used as a loading control.

After washing, membranes were incubated for 1 h at room temperature with HRP-conjugated secondary antibody (P0260; Dakocytomation, Agilent, Cernusco sul Naviglio, Italy), diluted 1:100,000 in blocking buffer. Signal detection was performed using the Westar Supernova system (Cyanagen, Bologna, Italy) and images were acquired with the iBright™ CL1000 platform (Thermo Fisher Scientific, Monza, Italy). iBright^TM^ Analysis software (v. 5.6.0) was used for semiquantitative analysis of the bands (Thermo Fisher Scientific, Monza, Italy). Molecular weights were estimated using a pre-stained marker (ab116028; Abcam, Cambridge, UK).

### 2.4. Immunofluorescence

Sub-confluent ATII-like cells grown on glass coverslips (25 × 25 mm) were used for immunofluorescence analysis of aquaporins. Cells were fixed with 4% paraformaldehyde for 10 min and stored in PBS at 4 °C until further processing. Antigen retrieval was performed by heating samples in citrate buffer (10 mM, pH 6.0) for 10 min.

Non-specific binding was blocked using 3% BSA in PBS for 30 min at room temperature. Samples were incubated overnight at 4 °C with primary antibodies (Table S2), followed by incubation with Alexa Fluor^®^ 488-conjugated secondary antibodies (115-547-003; 1:500 dilution; Jackson ImmunoResearch Europe, Ely, UK) for 30 min. Nuclei were counterstained with Hoechst 33,342 (cat. n. 14533; Merck, Milan, Italy).

Coverslips were mounted using FluoroSave™ reagent (345789; Merck, Milan, Italy), and images were acquired with a Leica TCS SP8 STED 3X confocal microscope (Leica biosystems, Buccinasco, Italy) equipped with a HC PL APO CS2 63x 1.4 NA oil-immersion objective. Negative controls included cells incubated with nonimmune serum (Figure S3).

### 2.5. HyPer7 Hydrogen Peroxide Indicators: Transfection and Detection of H_2_O_2_ in Cytoplasmic, Nuclear, and Mitochondrial Compartments

To investigate H_2_O_2_ dynamics in different intracellular compartments, three plasmids encoding the genetically encoded sensor HyPer7 were employed, targeting cytosolic (pCS2 + HyPer7-NES), mitochondrial (pCS2 + MLS-HyPer7), and nuclear (pCS2 + HyPer7-NLS) localization. These constructs were kindly provided by Vsevolod Belousov (IBCh, Moscow, Russia) and are available via Addgene (plasmid # 136467; http://n2t.net/addgene:136467 (accessed on 23 February 2026); RRID: Addgene_136467; plasmid # 136470; http://n2t.net/addgene:136470 (accessed on 23 February 2026); RRID: Addgene_136470; plasmid # 136468; http://n2t.net/addgene:136468 (accessed on 23 February 2026); RRID: Addgene_136468) [23].

Cells were transiently transfected at approximately 60–70% confluence in 2 mL culture dishes. For transfection, 2 μg plasmid DNA was diluted in JetOPTIMUS buffer (#717-60; Polyplus Transfection, Illkirch-Graffenstaden, France) and combined with 2 µL JetOPTIMUS DNA Transfection Reagent (#117-15) according to the manufacturer’s protocol. After 10 min incubation at room temperature, the mixture was added to cells in 2 mL Opti-MEM™ medium (Thermofisher Scientific, Monza, Italy). Following 4 h incubation at 37 °C, the medium was replaced with complete Ham’s F12, and experiments were performed 24 h post-transfection.

Intracellular H_2_O_2_ levels were monitored by measuring HyPer7 fluorescence changes using a ratiometric approach, as previously described [23]. Time-lapse imaging was carried out at 1–2 s intervals for 1 min using a Leica TCS SP8 DLS confocal microscope, with dual excitation at 420/490 nm and emission detection at 530 nm. Since ratiometric and single-wavelength measurements provided comparable results, subsequent analyses were conducted using single excitation (490 nm) [24].

Prior to imaging, cells were equilibrated for 10 min at room temperature in physiological buffer containing 140 mM NaCl, 5 mM KCl, 2 mM CaCl_2_, 1 mM MgCl_2_, 10 mM D-glucose, and 1 mM HEPES (pH 7.4). Fluorescence acquisition was performed using an Olympus BX41 microscope equipped with a 60× water-immersion objective (LUMPlanFI 60×/0.90 W; Olympus, Olympus Italia S.r.l., Segrate, Italy) and a DMK 33UP1300 CCD camera (The Imaging Source, Visionlink, Seregno, MB, Italy) with a frame rate of 10 frames per second. H_2_O_2_ was applied at a final concentration of 50 µM.

Image analysis was performed using ImageJ (v1.54f), and kinetic parameters were obtained by fitting fluorescence curves to a one-phase association model. The initial rate constant (k) and maximal fluorescence (Y_max_) were calculated using GraphPad Prism software (v. 4.00).

### 2.6. Antioxidant Enzyme Activity Assays

The activities of superoxide dismutase (SOD), catalase (CAT), and glutathione peroxidase (GPX) were quantified in cell lysates using commercially available kits (Cayman Chemical, Ann Arbor, MI, USA, 706002, 707002, and 703102, respectively) following the manufacturer’s instructions.

Following treatment, cells were washed with PBS, collected by scraping, and pelleted by centrifugation at 1000× *g* for 10 min. The resulting pellets were homogenized using an Omni 10010ST homogenizer according to the supplier’s protocols.

Protein concentration was determined using the Bradford assay [25] prior to enzymatic activity measurements.

### 2.7. Statistics

Data are presented as mean ± standard deviation (SD) unless otherwise specified, based on five independent experiments. For live-cell studies, fluorescence changes were quantified in more than 20 cells per condition per experiment. Statistical analyses were performed using one-way ANOVA followed by Tukey’s post hoc test, using GraphPad Prism software (version 4.00). Differences were considered statistically significant at *p* < 0.05.

## 3. Results

### 3.1. AQP1, AQP3, AQP4, AQP5, AQP6, AQP9, and AQP11 Are Expressed in ATII-Like Cells

To assess the effects of three different smoke extracts—CSE, e-cigarette vapor, and HTP extracts—on the oxidative status of ATII-like cells, we first examined the expression of aquaporins at both the mRNA and protein levels.

**Figure 1:**
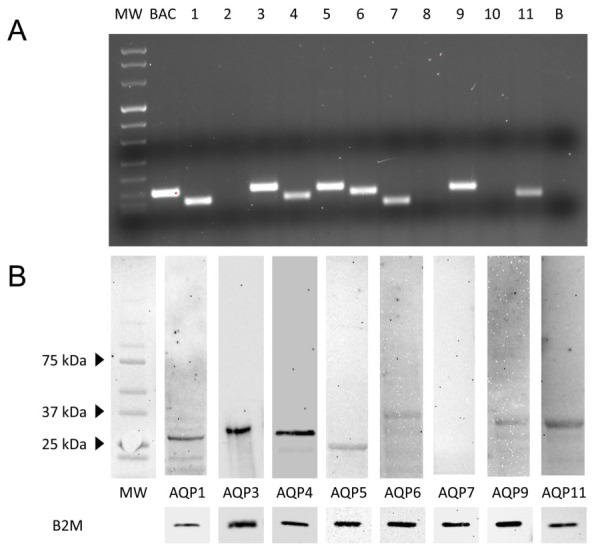
Expression of aquaporins in ATII-like cells. (**A**) Representative RT-PCR analysis of aquaporin mRNA expression. Lane numbers correspond to the different aquaporins analyzed. Amplicon sizes are reported in Table S1. β-actin (BAC) was used as housekeeping gene. (**B**) Representative immunoblot analysis of aquaporin protein expression (AQP1, AQP3, AQP4, AQP5, AQP6, AQP7, AQP9, AQP11). Molecular weights are indicated on the left and are consistent with published data. β2-microglobulin (B2M) was used as loading control. Data are representative of four independent experiments. MW, molecular weight marker; B, blank.

RT–PCR analysis performed on total RNA extracted from ATII-like cells revealed single bands of the expected sizes (Figure 1A). Eight of the eleven AQPs analyzed were expressed at the mRNA level: AQP1, AQP3, AQP4, AQP5, AQP6, AQP7, AQP9, and AQP11.

Western blot analysis was subsequently carried out to confirm AQP expression at the protein level. Protein bands corresponding to all detected AQPs were observed, with the exception of AQP7 (Figure 1B). The estimated molecular weights, calculated as described in the Section 2, were consistent with those reported in the literature. Expression of the housekeeping protein β2-microglobulin (B2M) was also confirmed (Figure 1B).

**Figure 2:**
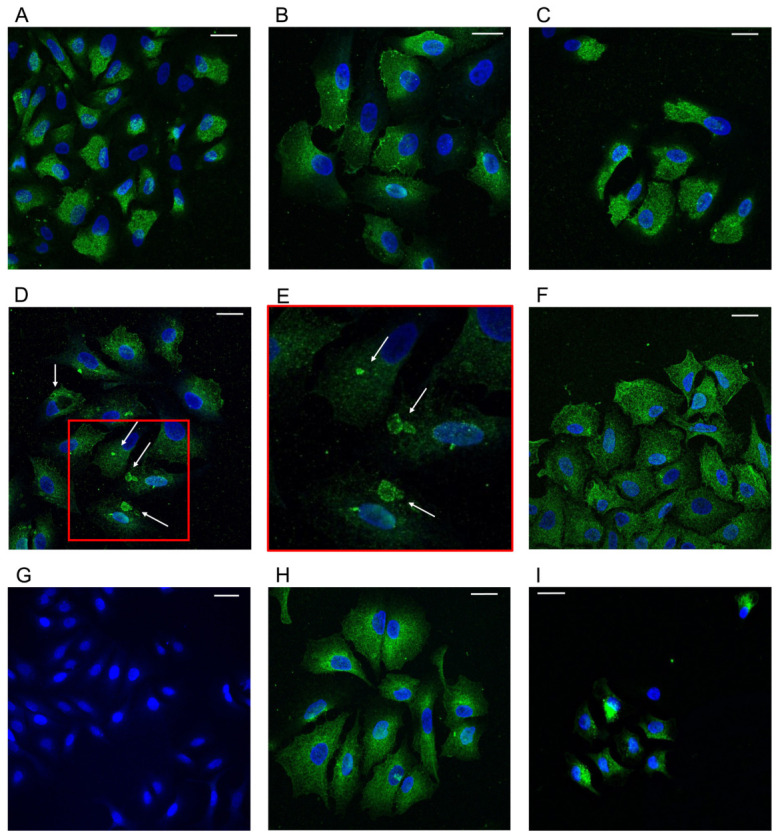
Immunolocalization of AQP1 (**A**), AQP3 (**B**), AQP4 (**C**), AQP5 ((**D**,**E**), inset in red), AQP6 (**F**), AQP7 (**G**), AQP9 (**H**), and AQP11 (**I**) in ATII-like cells. Representative confocal immunofluorescence images of AQP localization (green) at the plasma membrane and in intracellular compartments. Nuclei were counterstained with DAPI (blue). (**D**,**E**) Arrows indicate intracellular vesicles. Scale bars: 20 µm. Images are representative of four independent experiments.

The cellular localization of AQPs in ATII-like cells was further investigated by immunofluorescence analysis. As shown in Figure 2, AQP immunoreactivity was localized at the plasma membrane and in intracellular compartments. Additional AQP5 staining was also detected in the membranes of intracellular vesicles (Figure 2D,E). AQP11 displayed mainly cytosolic fluorescence, in agreement with previous reports describing its localization to the endoplasmic reticulum (Figure 2I) [17]. In line with the Western blot results, immunostaining for AQP7 was negative, confirming the absence of detectable protein expression (Figure 2G). No specific fluorescence was observed in negative control samples (Figure S3).

### 3.2. Cigarette Smoke and Heated Tobacco Product Extracts Strongly Affect AQP-Mediated Hydrogen Peroxide Permeability Across the Plasma Membrane

The effects of CSE, e-cigarette vapor, and HTP extracts on hydrogen peroxide diffusion across the plasma membrane of ATII-like cells were assessed using the cytosolic HyPer7-NES biosensor. Cells were exposed to each extract for 3 h, and HyPer7 fluorescence was monitored over time before and after the addition of H_2_O_2_.

**Figure 3:**
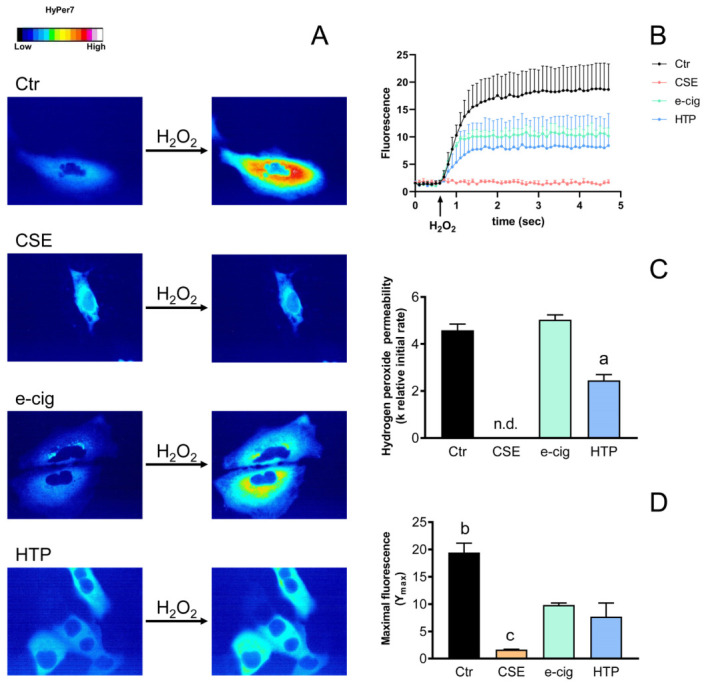
Effect of cigarette smoke extract (CSE), e-cigarette extract (e-cig), and heated tobacco product extract (HTP) on hydrogen peroxide diffusion across the plasma membrane of ATII-like cells. (**A**) Representative frames extracted from time-lapse videos showing the time course of HyPer7 fluorescence in untreated control cells (Ctr) and in cells treated with CSE, e-cig, or HTP. Left panels show cells before, and right panels after, the addition of 50 μM H_2_O_2_. The increase in HyPer7 fluorescence is displayed in pseudocolor, as indicated by the scale bar. (**B**) Time course of HyPer7 fluorescence in untreated and treated cells following the addition of 50 μM H_2_O_2_. Data are expressed as mean ± SD of five independent experiments. (**C**,**D**) Hydrogen peroxide permeability (k, relative initial rate; (**C**)) and maximal HyPer7 fluorescence (Y_max_; (**D**)) were obtained by fitting the experimental data from H_2_O_2_ time-course curves with a one-phase exponential association equation. a, *p* < 0.0003 compared to Ctr and e-cig; b, *p* < 0.002 compared to CSE, e-cig, and HTP; c, *p* < 0.0075 compared to e-cig (one-way ANOVA followed by Tukey post hoc test). n.d., not detectable.

Representative frames from time-lapse recordings are shown in Figure 3A, with images acquired before (left panels) and after (right panels) the addition of 50 μM H_2_O_2_ under control and treatment conditions. CSE exposure resulted in an almost complete inhibition of H_2_O_2_ permeability, as evidenced by the nearly absent increase in HyPer7 fluorescence following H_2_O_2_ addition (Figure 3B). Accordingly, the relative initial rate constant (k) was undetectable, and the maximal fluorescence response (Y_max_) was reduced by approximately 92% compared with control cells (Figure 3C,D). HTP treatment significantly reduced the relative initial rate constant (k) by approximately 50% compared with both control and e-cigarette-treated cells (Figure 3C). Consistently, Y_max_ values were reduced by about 60% and 50% in HTP and e-cigarette extract-treated cells, respectively, although these reductions were markedly less pronounced than those observed following CSE exposure (Figure 3D).

Overall, these results indicate that CSE almost completely abolishes H_2_O_2_ permeability across the plasma membrane, and HTP induces a substantial but partial reduction, whereas e-cigarette treatment exerts only minor effects.

### 3.3. Cigarette Smoke Extract Impairs AQP-Mediated Hydrogen Peroxide Permeability Across the Inner Mitochondrial Membrane

To determine whether CSE, e-cigarette extract, and HTP extract also affect H_2_O_2_ permeability across the inner mitochondrial membrane, experiments analogous to those described above were performed using a HyPer7 biosensor targeted to the mitochondrial matrix.

**Figure 4:**
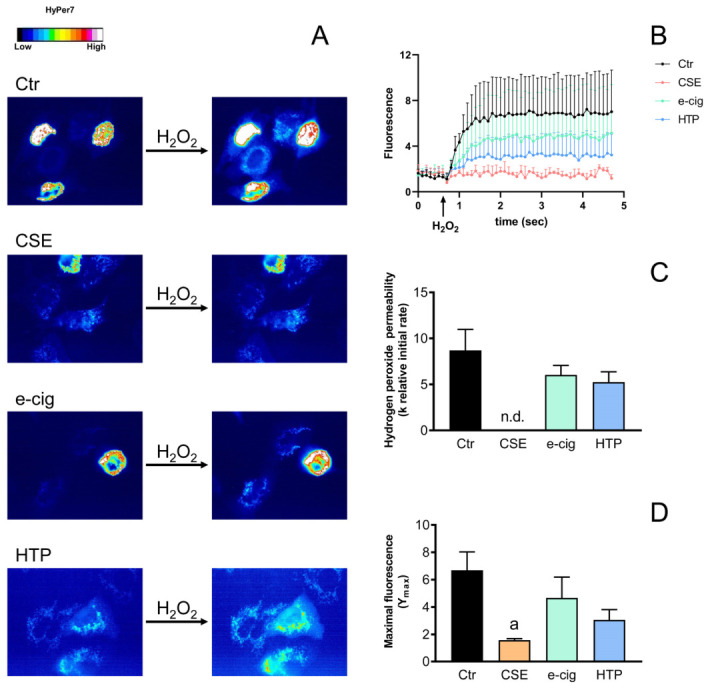
Effect of cigarette smoke extract (CSE), e-cigarette extract (e-cig), and heated tobacco product extract (HTP) on hydrogen peroxide diffusion across the inner mitochondrial membrane of ATII-like cells. (**A**) Representative frames extracted from time-lapse videos showing the time course of HyPer7 fluorescence in untreated control cells (Ctr) and in cells treated with CSE, e-cig, or HTP. Left panels show cells before, and right panels after, the addition of 50 μM H_2_O_2_. The increase in HyPer7 fluorescence is displayed in pseudocolor, as indicated by the scale bar. (**B**) Time course of HyPer7 fluorescence in untreated and treated cells following the addition of 50 μM H_2_O_2_. Data are expressed as mean ± SD of five independent experiments. (**C**,**D**) Hydrogen peroxide permeability (k, relative initial rate; (**C**)) and maximal HyPer7 fluorescence (Y_max_; (**D**)) were obtained by fitting the experimental data from H_2_O_2_ time-course curves with a one-phase exponential association equation. a, *p* < 0.02 compared to Ctr (one-way ANOVA followed by Tukey post hoc test). n.d., not detectable.

Consistent with the observations at the plasma membrane, CSE treatment almost completely suppressed H_2_O_2_ permeability across the inner mitochondrial membrane, as indicated by the lack of a detectable fluorescence increase following H_2_O_2_ addition (Figure 4A,B). Under these conditions, the relative initial rate constant (k) was virtually undetectable, and Y_max_ was reduced by approximately 77% compared with control cells (Figure 4C,D).

In contrast, neither HTP nor e-cigarette treatment induced significant changes in either the rate constant or maximal fluorescence response.

Taken together, these findings demonstrate that CSE profoundly impairs AQP-mediated H_2_O_2_ permeability across the inner mitochondrial membrane, whereas HTP and e-cigarette extract exposure do not produce detectable effects under the conditions tested.

### 3.4. Cigarette Smoke Extract and Heat Tobacco Product Extract Impair Hydrogen Peroxide Permeability Across the Nuclear Envelope

**Figure 5:**
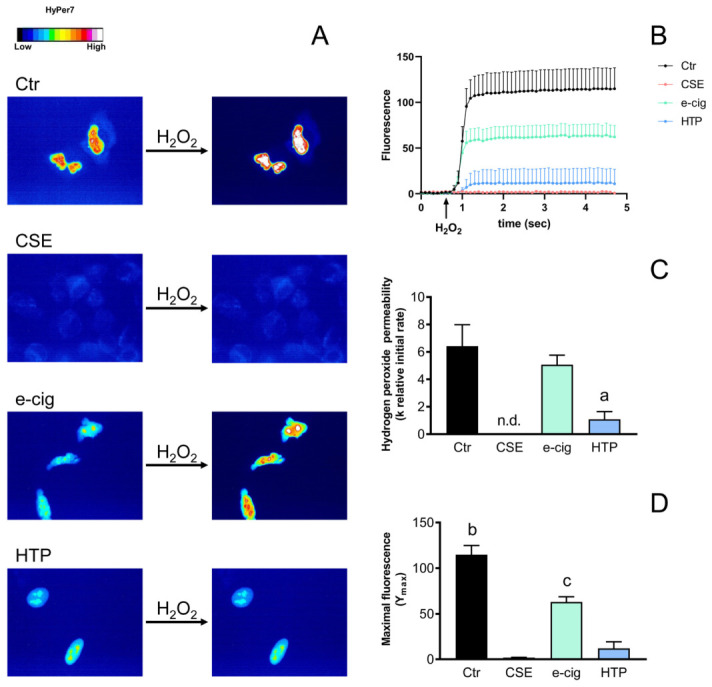
Effect of cigarette smoke extract (CSE), e-cigarette extract (e-cig), and heated tobacco product extract (HTP) on hydrogen peroxide diffusion across the nuclear membrane of ATII-like cells. (**A**) Representative frames extracted from time-lapse videos showing the time course of HyPer7 fluorescence in untreated control cells (Ctr) and in cells treated with CSE, e-cig, or HTP. Left panels show cells before, and right panels after, the addition of 50 μM H_2_O_2_. The increase in HyPer7 fluorescence is displayed in pseudocolor, as indicated by the scale bar. (**B**) Time course of HyPer7 fluorescence in untreated and treated cells following the addition of 50 μM H_2_O_2_. Data are expressed as mean ± SD of five independent experiments. (**C**,**D**) Hydrogen peroxide permeability (k, relative initial rate; (**C**)) and maximal HyPer7 fluorescence (Y_max_; (**D**)) were obtained by fitting the experimental data from H_2_O_2_ time-course curves with a one-phase exponential association equation. a, *p* < 0.05 compared to Ctr and e-cig; b, *p* < 0.0003 compared to CSE, e-cig, and HTP; c, *p* < 0.0004 compared to CSE, and HTP (one-way ANOVA followed by Tukey post hoc test). n.d., not detectable.

Cells expressing the nuclear-targeted HyPer7 biosensor were used to evaluate the effects of CSE, e-cigarette, and HTP extracts on H_2_O_2_ permeability across the nuclear envelope. Consistent with the observations at the plasma and mitochondrial membranes, CSE treatment almost completely abolished H_2_O_2_ permeability, as indicated by the absence of fluorescence increase following H_2_O_2_ addition (Figure 5A,B). Accordingly, both the relative initial rate constant (k) and the maximal response (Y_max_) were virtually undetectable (Figure 5C,D). HTP extract significantly reduced both k and Y_max_ values by approximately 83% and 88%, respectively, whereas e-cigarette extract did not produce significant changes (Figure 5C,D).

Overall, CSE and HTP markedly impaired H_2_O_2_ permeability across the nuclear envelope, while e-cigarette extract did not exert detectable effects.

### 3.5. Effects of Cigarette Smoke, Heat Tobacco Products, and E-Cigarette Extracts on Antioxidant Enzyme Activities

The activities of superoxide dismutase (SOD), catalase (CAT), and glutathione peroxidase (GPX) were measured in homogenates of ATII-like cells following 3 h exposure to CSE, e-cigarette, or HTP extracts.

**Figure 6:**
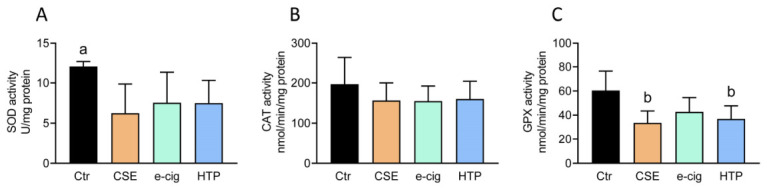
Effects of cigarette smoke extract (CSE), e-cigarette extract (e-cig), and heated tobacco product extract (HTP) on superoxide dismutase (SOD; (**A**)), catalase (CAT; (**B**)), and glutathione peroxidase (GPX; (**C**)) activities in ATII-like cells. SOD activity is expressed as U/mg protein, whereas CAT and GPX activities are expressed as nmol/min/mg protein. Data are presented as mean ± SD (N = 7). Statistical analysis was performed by one-way ANOVA followed by Tukey’s post hoc test. a, *p* < 0.05 vs. CSE, e-cig, and HTP; b, *p* < 0.02 vs. control (Ctr).

Exposure to CSE, e-cigarette, and HTP extracts significantly reduced SOD activity compared with controls, by approximately 50%, 38%, and 38%, respectively (Figure 6A). Catalase activity was largely unchanged, showing only a non-significant decrease of approximately 20% in all treatment groups (Figure 6B). GPX activity was significantly reduced following exposure to CSE and HTP extracts, whereas e-cigarette extract did not significantly affect GPX activity (Figure 6C).

## 4. Discussion

The generation of reactive oxygen species and the resulting oxidative stress represent early hallmarks of tissue damage induced by traditional cigarette smoking and play a central role in the development of COPD and lung cancer [1]. Given the difficulty of reducing cigarette consumption or achieving smoking cessation, alternative products marketed as potentially less harmful have been introduced. In the present study, we compared the effects of traditional cigarette smoke with two such alternatives—e-cigarettes and heated tobacco products—on a key cellular antioxidant system, namely peroxiporins.

Peroxiporins, a subgroup of aquaporins (AQPs), facilitate the diffusion of hydrogen peroxide (H_2_O_2_), the principal diffusible ROS, from the cell, thereby contributing to ROS elimination and preventing the onset of oxidative stress. As an experimental model, we used A549 cells differentiated into cells resembling type II pneumocytes (ATII-like cells), a well-established in vitro model for alveolar epithelial biology [19,20].

ATII-like cells were first characterized with respect to AQP expression. Eight AQPs were detected at the transcript level, and seven were confirmed at the protein level (Figure 1 and Figure 2). With the exception of AQP4, all expressed AQPs are known to be permeable to H_2_O_2_ and therefore functionally belong to the peroxiporin subclass. We then investigated the effect of traditional cigarette smoke extract on H_2_O_2_ diffusion across both the plasma membrane, the inner mitochondrial membrane, and the nuclear envelope. Our working hypothesis was that CSE could exacerbate oxidative stress not only by increasing ROS production, but also by inhibiting H_2_O_2_ diffusion from the cell and from mitochondria to the cytosol. Consistent with this hypothesis, CSE treatment profoundly impaired AQP-dependent H_2_O_2_ permeability across both membranes (Figure 3 and Figure 4). A second major finding of this study is that exposure to heated tobacco products extract significantly reduced H_2_O_2_ permeability across the plasma membrane, although to a lesser extent than CSE, while leaving mitochondrial permeability largely unaffected (Figure 3 and Figure 4). In contrast, e-cigarette exposure exerted little or no effect on AQP-mediated H_2_O_2_ diffusion. Lastly, CSE and HTP extract dramatically reduced H_2_O_2_ diffusion through the nuclear membrane (Figure 5). To rule out the possibility that the changes in H_2_O_2_ permeability induced by CSE, e-cig, and HTP were due to alterations in AQP expression, AQP transcript levels were assessed by real-time RT-PCR. No significant differences in transcript expression were observed (Figure S4). Consistently, Western blot analysis of three randomly selected AQPs confirmed these findings at the protein level (Figure S5).

Previous studies have shown that cigarette smoke and CSE consistently alter AQP expression and function in the lung, thereby affecting epithelial barrier integrity, mucus production, inflammation, and long-term lung function. In murine models, cigarette smoke exposure induces COPD-like features, including inflammation, cytokine release, increased pulmonary permeability, and oxidative stress, in parallel with reduced AQP1 expression [26]. Notably, oral administration of the flavonoid naringin restored AQP1 expression and tight junction integrity, reducing endothelial hyperpermeability and inflammation; these protective effects were absent in AQP1 knockout mice, highlighting a causal role for AQP1. Interestingly, the aglycone naringenin has been shown to exert antioxidant effects by modulating peroxiporin pore gating [22].

Additional evidence indirectly supports the link between cigarette smoke exposure and AQP dysregulation. CSE-induced proinflammatory changes in the lung microbiome have been associated with reduced expression of AQP1, surfactant protein A (SP-A), and other mucosal defense proteins [27]. Similarly, comparative analyses of lung toxicity models revealed significant downregulation of AQP4 and SP-A transcripts following chronic CSE exposure in mice [28]. In humans, cigarette smoke exposure has also been shown to reduce AQP5 expression and to correlate with impaired lung function [29]. While these studies clearly demonstrate AQP downregulation in response to cigarette smoke, the functional consequences of such changes have remained incompletely understood.

The present study demonstrates that, in addition to reduced AQP expression, CSE profoundly disrupts AQP-mediated H_2_O_2_ permeability, thereby severely impairing ROS diffusion and exacerbating oxidative stress. In this context, it is noteworthy that a 3 h CSE exposure significantly reduced the activity of SOD and GPX antioxidant enzymes, further worsening the oxidative status of ATII-like cells (Figure 6). Consistent with our findings, nine-week cigarette smoke extract injection has been shown to impair SOD, CAT, and GPX activity in whole lung homogenates from BALB/c mice [30]. Loss of these enzymatic defenses markedly increases susceptibility to CSE-induced cellular injury.

In addition to CSE, exposure to HTP and e-cigarette extracts altered antioxidant enzyme activity (Figure 6). Specifically, SOD activity was reduced following exposure to all tested extracts, whereas GPX activity was significantly reduced only after CSE and HTP extract exposure. In contrast, CAT activity was not significantly affected under our experimental conditions. This observation is consistent with previous studies reporting nicotine-driven oxidative stress in lung and non-lung tissues, closely associated with alterations in first-line antioxidant enzymes [31,32,33,34]. The results reported here are not fully consistent with previously published findings. In our experimental conditions, CAT activity was not significantly altered by any of the tested extracts, and GPX activity was unaffected by e-cigarette extract treatment. These discrepancies may be explained by differences in exposure duration. Notably, our protocol involved acute treatment, whereas the studies cited above employed substantially longer exposure periods, ranging from 4 to 22 weeks in vivo and from 24 h to 21 days in vitro [28,29,30,31,32]. Thus, the lack of significant modulation of CAT and—for e-cigarette extract only—of GPX activity in our model may reflect an early-phase response to oxidative challenge, preceding the enzyme adaptations typically observed following chronic exposure.

Cigarette smoke is widely recognized as a major contributor to COPD development, in part through the induction of mitochondrial dysfunction [35,36]. CSE has been shown to alter mitochondrial membrane permeability, increase mitochondrial ROS production, and trigger apoptosis [37,38]. In the present study, we specifically examined H_2_O_2_ permeability across the inner mitochondrial membrane, hypothesizing that cigarette smoke components might impair mitochondrial ROS elimination. Our results clearly demonstrate that CSE almost completely abolishes mitochondrial H_2_O_2_ diffusion, whereas HTP and e-cigarette extract exposure do not produce detectable effects (Figure 4). This finding aligns with evidence showing that concentrations of most harmful compounds are substantially lower in HTP aerosols than in conventional cigarette smoke, but higher than in e-cigarette emissions [39].

Since CSE is known to induce nuclear DNA damage [40,41], we investigated whether CSE, e-cigarette extract, and HTP extract affect H_2_O_2_ permeability across the nuclear envelope. Our results show that both CSE and HTP extract markedly reduced H_2_O_2_ permeability at the nuclear level (Figure 5), suggesting a potential interference with redox signaling between the cytoplasm and nucleus.

Although aquaporins are primarily characterized as plasma membrane water channels, specific isoforms have been reported to localize to the nuclear envelope or perinuclear region [42,43]. However, the available evidence remains limited and largely cell type-specific, and their proposed functions do not necessarily overlap with classical roles in nuclear volume regulation. Indeed, passive water diffusion into the nucleus is generally thought to occur predominantly through the nuclear pore complex.

Given the physicochemical similarities between H_2_O and H_2_O_2_, it is currently not possible to determine whether the inhibitory effects of CSE and HTP at the nuclear membrane level involve aquaporins (peroxiporins), the nuclear pore complex, or both. Furthermore, nuclear localization of aquaporins is not yet considered a universally established feature of the aquaporin family, and their specific transport properties, regulatory mechanisms, and functional relevance across different cell types remain incompletely characterized.

Overall, converging evidence from chemical analyses, cellular studies, animal models, and short-term human investigations supports a hierarchy of lung toxicity—conventional cigarettes > heated tobacco products > e-cigarettes—although none of these products can be considered harmless [44,45,46,47,48]. Despite the limitation that our findings were obtained in an ATII-like cell model rather than primary human lung cells, the relative effects of CSE, HTP extract, and e-cigarette extract on plasma, mitochondrial, and nuclear membrane H_2_O_2_ permeability observed here are fully consistent with this toxicity gradient.

Conventional smoking drives oxidative stress through multiple, partially overlapping mechanisms. Cigarette smoke directly introduces ROS and free radicals into the lung, while smoke constituents react with biomolecules to generate additional ROS [49]. Moreover, smoke exposure activates lung macrophages, neutrophils, epithelial cells, and ROS-generating enzymes such as NADPH oxidases, further amplifying oxidative burden [49,50]. At the same time, antioxidant systems—including GPX, SOD, and CAT—are depleted or downregulated in smokers, aggravating redox imbalance (Figure 6; [49,50,51,52]).

This study has some limitations that should be acknowledged. First, the experiments were conducted using A549-derived ATII-like cells, which, although widely used as an in vitro model, do not fully recapitulate the complexity and heterogeneity of alveolar epithelium. In addition, the exposure conditions employed in this study were acute and therefore do not reflect the chronic exposure associated with long-term smoking or vaping, which may lead to adaptive or cumulative effects on oxidative stress pathways.

Furthermore, while our data support the involvement of aquaporin-mediated H_2_O_2_ diffusion, the specific contribution of individual aquaporin isoforms was not directly assessed, and the potential involvement of alternative transport mechanisms cannot be excluded. Future investigations utilizing genetic silencing approaches, such as siRNA knockdown, will be necessary to pinpoint the precise role of specific aquaporin isoforms involved in this process. Finally, this study primarily focuses on oxidative stress-related processes and does not address downstream biological consequences such as inflammation, epithelial barrier dysfunction, or long-term cellular damage. Future studies employing primary human lung cells, more complex airway models, in vivo systems, and chronic exposure paradigms will be important to validate and extend these findings.

Our study identifies an additional, previously underappreciated mechanism contributing to smoking-induced oxidative stress: the inhibition of AQP-mediated H_2_O_2_ transport. By impairing peroxiporin function at both the plasma membrane and mitochondrial levels, cigarette smoke limits cellular H_2_O_2_ diffusion, thereby exacerbating oxidative damage and mitochondrial dysfunction. However, the inhibitory effect of CSE and HTP extracts on H_2_O_2_ permeability across the nuclear envelope represents an unexpected and mechanistically intriguing finding that warrants further investigation.

## 5. Conclusions

In conclusion, this study demonstrates that short-term exposure to CSE causes a marked impairment of H_2_O_2_ diffusion in ATII-like cells, associated with alterations in antioxidant enzyme activity and a profound inhibition of H_2_O_2_ diffusion from mitochondria. HTP extract exerted detectable detrimental effects by reducing plasma membrane H_2_O_2_ permeability and modulating antioxidant enzyme activity, while not significantly affecting mitochondrial H_2_O_2_ permeability. Both CSE and HTP also abolished the H_2_O_2_ permeability of the nuclear envelope. In contrast, e-cigarette extract exposure produced only minor effects on plasma membrane H_2_O_2_ diffusion and did not alter mitochondrial and nuclear H_2_O_2_ permeability. Notably, SOD activity was reduced following exposure to all tested extracts, whereas GPX activity was reduced only after CSE and HTP treatment.

**Figure 7:**
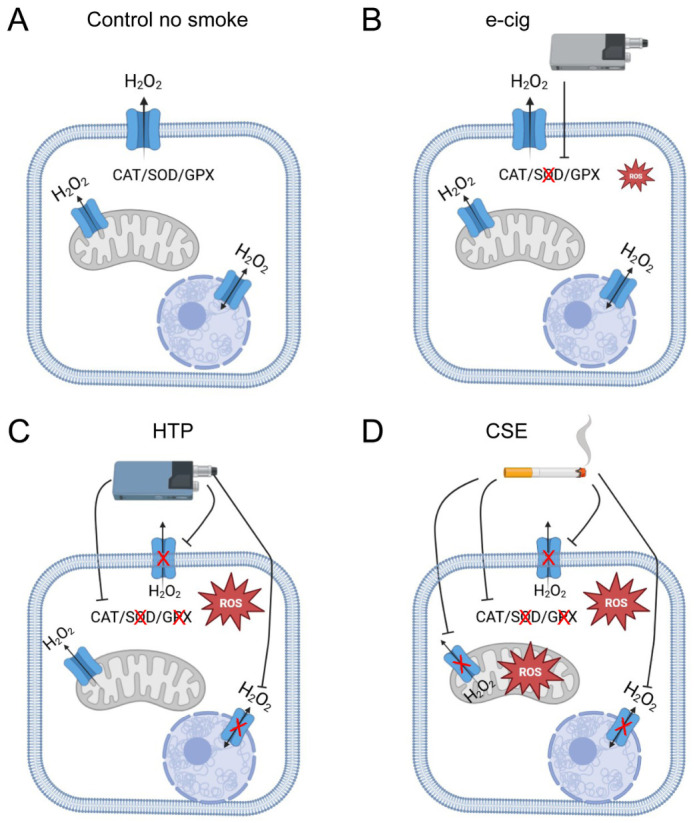
A schematic model of the effect of cigarette smoke extract (CSE; (**D**)), e-cigarette extract (e-cig; (**B**)), and heated tobacco product extract (HTP; (**C**)) treatment on activities of antioxidant enzymes catalase (CAT), superoxide dismutase (SOD), and glutathione peroxidase (GPX), and on hydrogen peroxide diffusion across the plasma, nuclear, and inner mitochondrial membranes of ATII-like cells. Condition in untreated controls is also shown (**A**).

The main findings of this study are summarized in Figure 7 and further support the importance of smoking cessation or reduction as an effective strategy to limit oxidative stress and its long-term pathological consequences. Although alternative nicotine delivery systems display a lower toxicity profile than conventional cigarettes, our results indicate that their biological effects are not negligible and should be carefully considered in the context of long-term exposure.

## Data Availability

The original contributions presented in this study are included in the article/Supplementary Materials. Further inquiries can be directed to the corresponding author.

## Supplementary Materials

The following supporting information can be downloaded at https://www.mdpi.com/article/10.3390/cells15121112/s1: Table S1: Primer sequences used for reverse transcription/polymerase chain reaction. Table S2: Antibodies used for immunoblotting and immunofluorescence assays. Figure S1: Characterization of A549-derived alveolar type II-like (ATII-like) cells. Figure S2: Effect of cigarette smoke extract (CSE), e-cigarette extract (e-cig), and heated tobacco product extract (HTP) on ATII-like cell viability (A) and number (B). Figure S3: Immunofluorescence negative control in ATII-like cells. Figure S4: Effect of cigarette smoke extract (CSE), e-cigarette extract (e-cig), and heated tobacco products extract (HTP) on AQP mRNA expression in ATII-like cells. Figure S5: Effects of cigarette smoke extract (CSE), e-cigarette extract (e-cig), and heated tobacco product extract (HTP) on aquaporin protein expression (AQP1, AQP3, and AQP6) in ATII-like cells.
