# Supplementary material for: Combustion and Heated Tobacco Cigarettes, but Not E-Cigarettes, Impair Aquaporin-Dependent H2O2 Permeability in ATII-Like Cells
Source: Cells. 2026 Jun 19;15(12):1112. doi: 10.3390/cells15121112 (PMC13297388; doi:10.3390/cells15121112)
Supplement: Supplementary file 1 [file cells-15-01112-s001.zip › cells-4197376-supplementary.pdf]

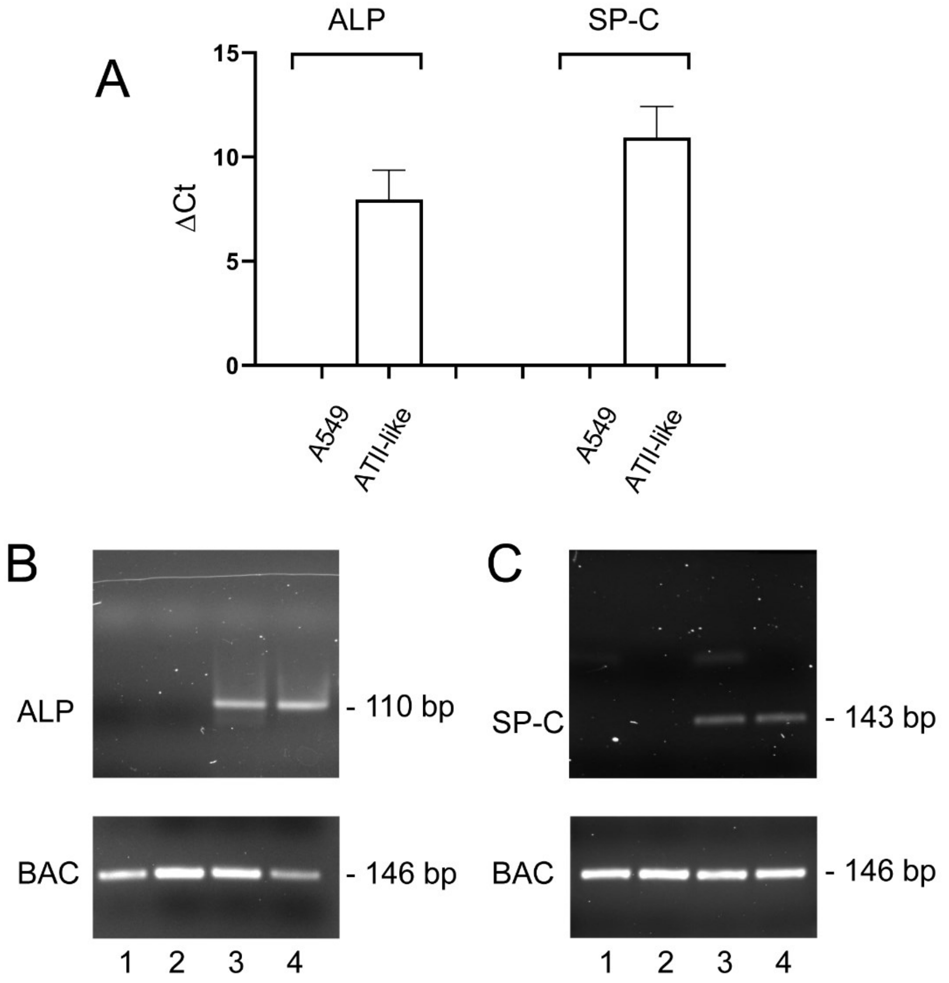

Figure S1. Characterization of A549-derived alveolar type II-like (ATII-like) cells. (A) Quantitative RT-PCR analysis of alveolar epithelial cell markers in A549 and ATII-like cells. The expression of alkaline phosphatase (ALP) and surfactant protein C (SP-C) is reported as ΔCt values. Data are presented as mean ± SD. (B) Representative RT-PCR analysis of ALP expression showing the expected 110-bp amplicon. (C) Representative RT-PCR analysis of SP-C expression showing the expected 143-bp amplicon. In panels B and C, β-actin (BAC, 146 bp) was used as the internal control. Lanes 1–2 correspond to A549 cells, whereas lanes 3–4 correspond to ATII-like cells.

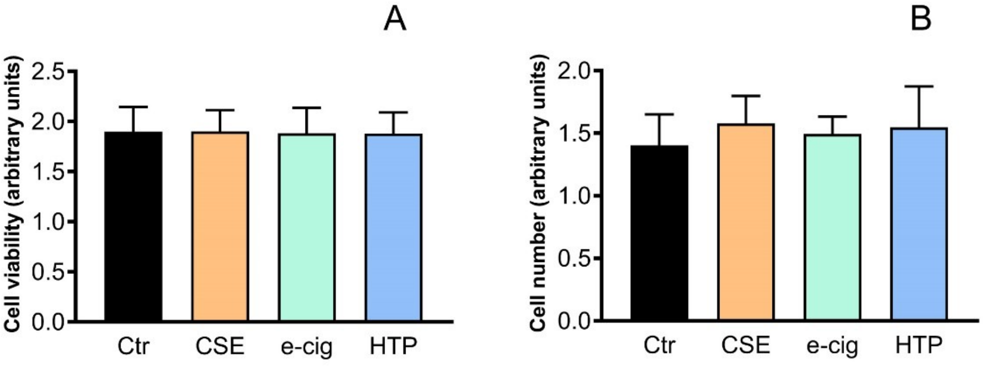

Figure S2. Effect of cigarette smoke extract (CSE), e-cigarette extract (e-cig), and heated tobacco products extract (HTP) on ATII-like cell viability (**A**) and number (**B**). Cell viability was determined by Cell Counting Kit-8 (MedChem Express). Cell number was determined by cristal violet staining. Data are expressed as mean ± S.E.M. N = 16.

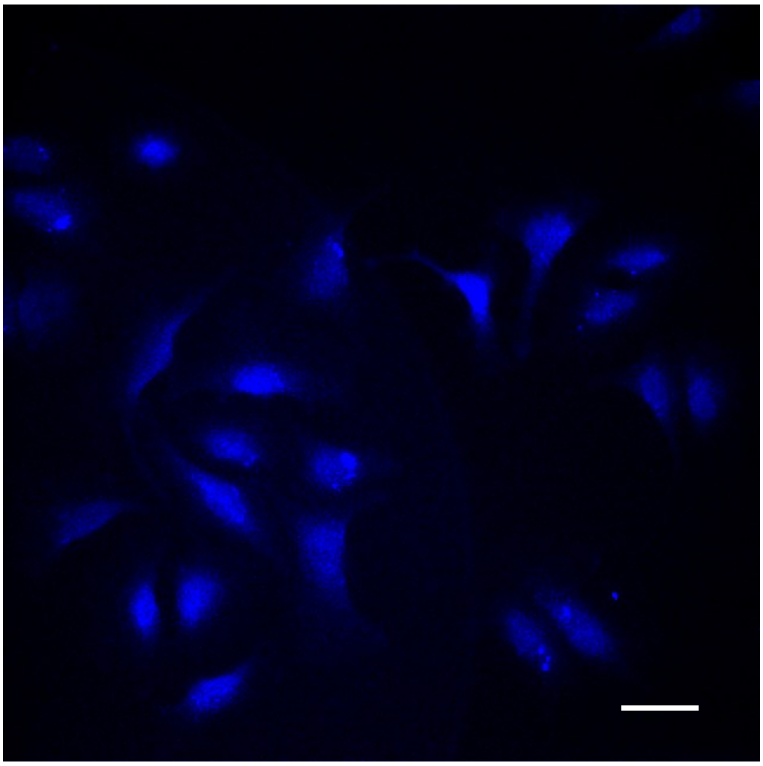

Figure S3. Immunofluorescence negative control in ATII-like cells. No specific staining was observed when anti-aquaporin antibodies were replaced with preimmune serum. Cell nuclei were counterstained with DAPI (blue). Scale bar = 20 μm.

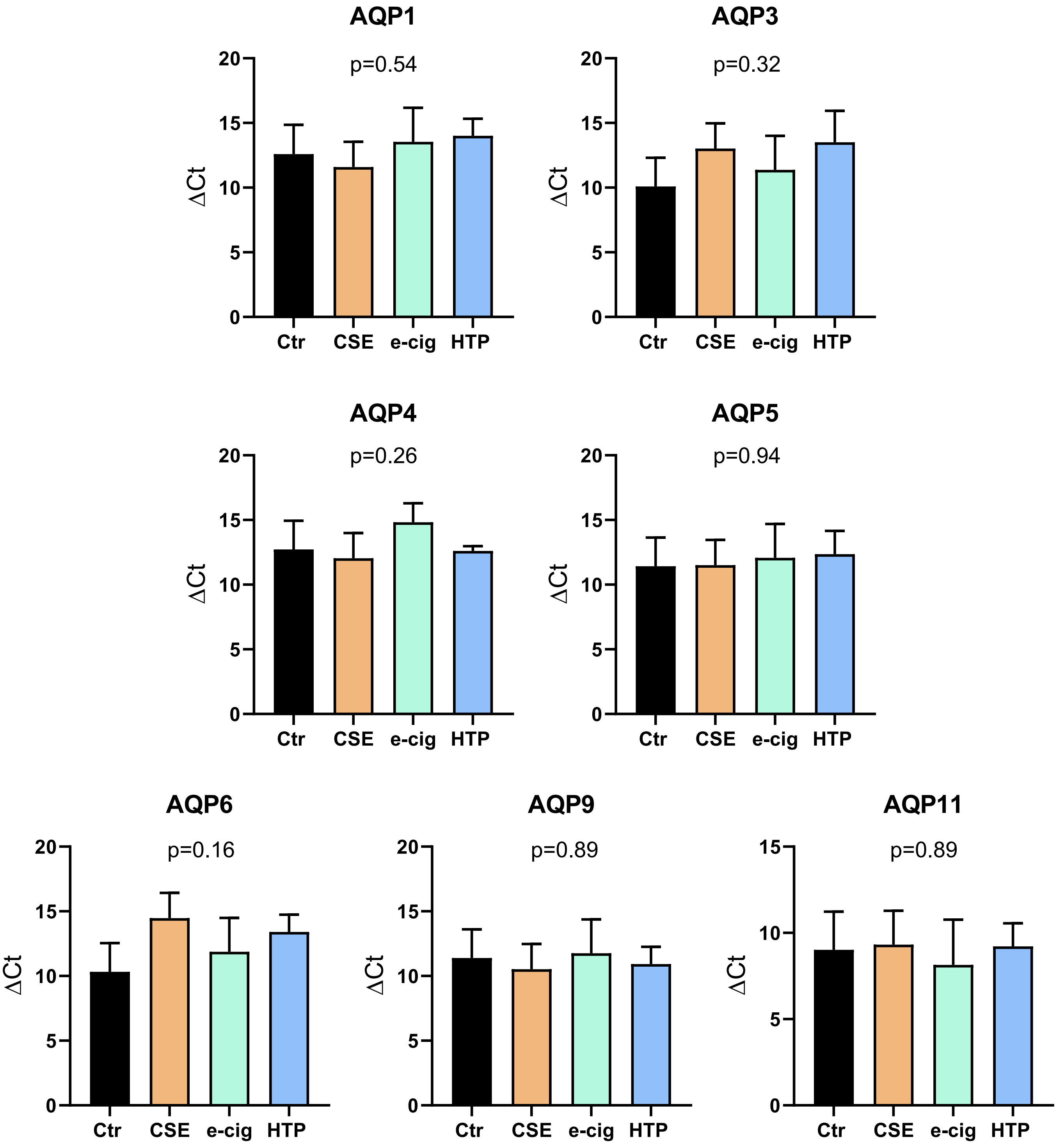

Figure S4. Effect of cigarette smoke extract (CSE), e-cigarette extract (e-cig), and heated tobacco products extract (HTP) on AQP mRNA expression in ATII-like cells. AQP mRNA levels were measured by real-time RT-PCR and normalized to the housekeeping gene β-actin. Bars represent the mean ± SD of ΔCt values obtained from four independent experiments, each performed using different RNA samples. mRNA levels were not significantly different among the four groups; p-values are shown. (ANOVA followed by Tukey's post hoc test).

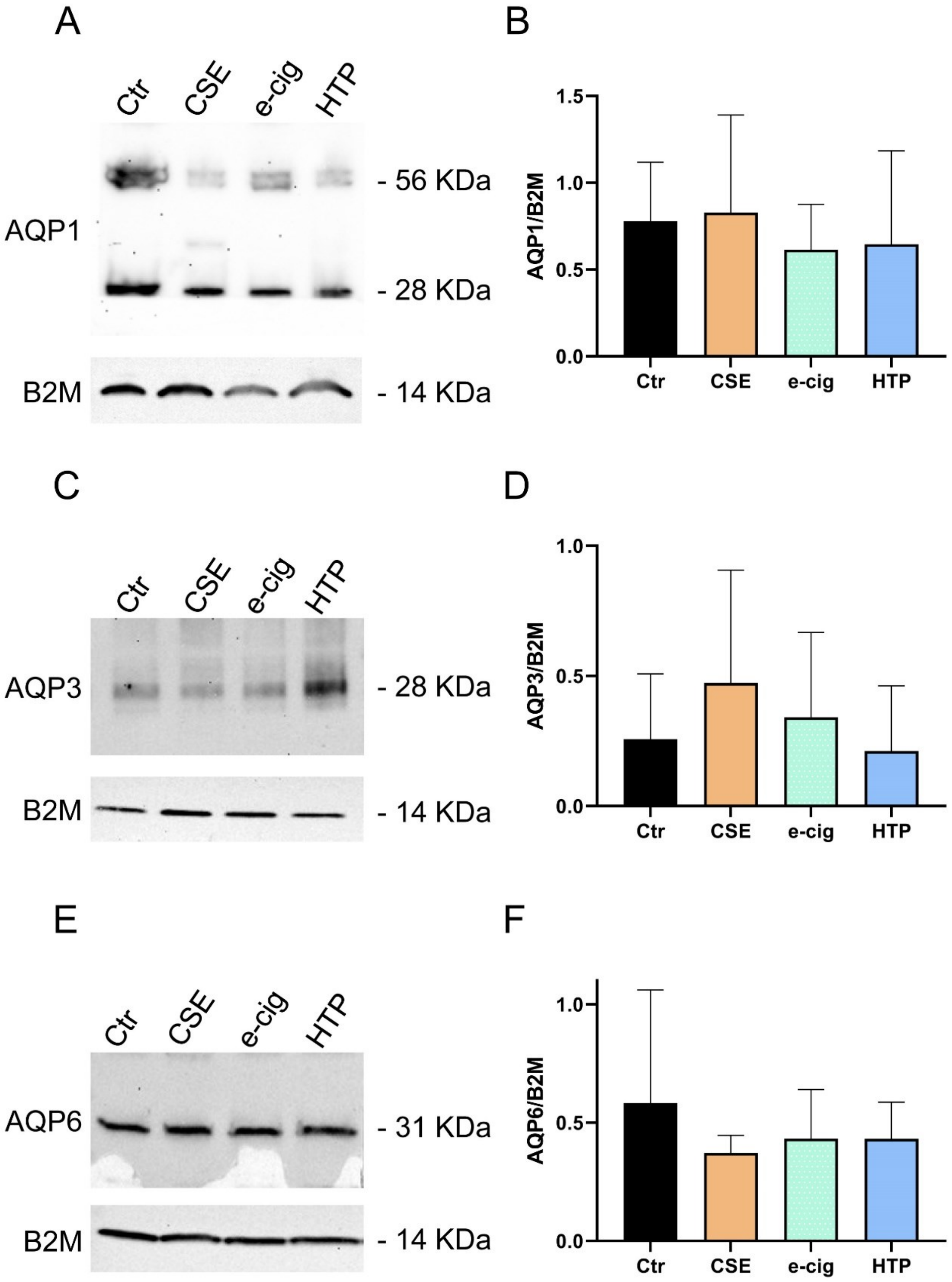

Figure S5. Effects of cigarette smoke extract (CSE), e-cigarette extract (e-cig), and heated tobacco product extract (HTP) on aquaporin protein expression (AQP1, AQP3, and AQP6) in ATII-like cells. (A, C, E) Representative immunoblots of AQP1, AQP3, AQP6, and β2-microglobulin (B2M) are shown. Molecular weights are indicated on the right. (B, D, F) Band densitometric analysis was performed using iBright Analysis Software (Thermo Fisher Scientific, Milan, Italy) and values were normalized to B2M expression. Bars represent means ± SD of four independent experiments.

Table S1. Primer sequences used for reverse transcription/polymerase chain reaction.

Gene	Primer sequences		Ta (°C)	Size (bp)	Accession number
AQP1	F	5′-ACACCTCCTGGCTATTGACTACAC-3′	62	134	NM_198098.3; variants 1, 5
	R	5′-CCGATGAATGGCCCCACCCAGAA -3′			
AQP2	F	5′-CACCTCCTTGGGATCCATTACACC-3′	62	95	NM_000486.5
	R	5′-ACCCAGTGGTCATCAAATTTGCC-3′			
AQP3	F	5′-CCTGGTGATGTTTGGCTGTGGCTC-3′	62	147	NM_004925; variants 1, 2
	R	5′-TTCAGGTGGGCCCCAGAGACC-3′			
AQP4	F	5′-GGAGTCACCATG GTTCATGGAA-3′	60	123	NM_001650.6; variants 1-3, X1
	R	5′-AGTGACATCAGTCCGTTTGGAA-3′			
AQP5	F	5′-GGTGGTGGAGCTGATTCTGA-3′	58	142	NM_001651.3; variant X1
	R	5′-GAAGTAGATTCCGACAAGGTGG-3′			
AQP6	Hs_AQP6_1_SG QuantiTect Primer Assay QT00010633, Qiagen		60	129	NM_ 001652, XM_006719375
AQP7	F	5′-GGACAGCTGATGGTGACCGG-3′	62	104	NM_001170.2; variants 1-4, X1-X15
	R	5′-AGCCACGCCTCATTCAGGAA-3′			
AQP8	F	5′-TGGAGAGATAGCCATGTGTGAG-3′	60	106	NM_001169; variants X1,X2
	R	5′-TGGCTGCACAAACCGTTCGT-3′			
AQP9	F	5′-CCCAGCTGTGTCTTTAGCAA-3′	58	133	NM_020980.4; variants 1,2,3
	R	5′-AAGTCCATCATAGTAAATGCCAAA-3′			
AQP10	F	5′-CCTATGTTCTCTACCATGATGCCC-3′	60	137	NM_080429
	R	5′-CTGATCCAGGAAGCCATTGTTC-3′			
AQP11	F	5′-TTTCTCTTCCACAGCGCTCT-3′	58	115	NM_173039; variant 1
	R	5′-CTCCTGTTAGACTTCCTCCTGC-3′			
SP-C	F	5′-CTGGTTACCACTGCCACCTT-3′	60	143	NM_003018.4; variants 1-14
	R	5′- TCAAGACTGGGGATGCTCTC-3′			
ALP	Hs_ALPL_1_SG QuantiTect Primer Assay QT00012957, Qiagen		60	110	NM_000478, NM_001127501, NM_001177520
BAC	Hs_ACTB_1_SG QuantiTect Primer Assay QT00095431, Qiagen		60	146	NM_001101

Forward, F; Reverse, R; Annealing temperature, Ta; surfactant protein C, SP-C; alkaline phosphatase, ALP; β-actin, BAC.

Table S2. Antibodies used for immunoblot and immunofluorescence assays.

Antibody	Host	Source (catalog)	WB dilution	IF dilution
Anti-AQP1	Rb	St John's Laboratory Ltd, London, U.K. (STJ117224)	1:1000	1:100
Anti-AQP3	Rb	Life Technologies Italia, Monza, Italy (PA577840)	1:2500	1:400
Anti-AQP4	Rb	St John's Laboratory Ltd, London, U.K. (STJ22661)	1:500	1:100
Anti-AQP5	Rb	Merck, Milan, Italy (A4985)	1:1000	1:100
Anti-AQP6	Rb	Alpha Diagnostic International, San Antonio, U.S.A. (AQP61-A)	1:3000	1:500
Anti-AQP7	Rb	Abcam, Cambridge, U.K. (ab32826)	1:1000	1:250
Anti-AQP8	Rb	Merck, Milan, Italy (HPA046259)	1:1000	-
Anti-AQP9	Rb	Life Technologies Italia, Monza, Italy (PA5-97110)	1:500	1:200
Anti-AQP11	Rb	BOSTER biological technology, California, U.S.A. (PB10044)	1:1000	1:100
Anti-B2M	Rb	Abcam, Cambridge, U.K. (ab75853)	1:10000	-

Western blot, WB; Immunofluorescence, IF; Rabbit, Rb; β2-microglobulin, B2M.
